# Convergent Validity of Ratings of Perceived Exertion During Resistance Exercise in Healthy Participants: A Systematic Review and Meta-Analysis

**DOI:** 10.1186/s40798-021-00386-8

**Published:** 2022-01-08

**Authors:** John W. D. Lea, Jamie M. O’Driscoll, Sabina Hulbert, James Scales, Jonathan D. Wiles

**Affiliations:** 1 School of Psychology and Life Sciences, Canterbury Christ Church University, Kent, CT1 1QU UK; 2 Institute of Population and Health Sciences, Queen Mary University of London, London, E1 4NS UK

**Keywords:** Exercise intensity, Physiological exertion, RPE, Workload, Strength training

## Abstract

**Background:**

The validity of ratings of perceived exertion (RPE) during aerobic training is well established; however, its validity during resistance exercise is less clear. This meta-analysis used the known relationships between RPE and exercise intensity (EI), heart rate (HR), blood lactate (BLa), blood pressure (BP) and electromyography (EMG) to determine the convergent validity of RPE as a measure of resistance exercise intensity and physiological exertion, during different forms of resistance exercise. Additionally, this study aims to assess the effect of several moderator variables on the strength of the validity coefficients, so that clearer guidance can be given on the use of RPE during resistance exercise.

**Methods:**

An online search of 4 databases and websites (PubMed, Web of Science SPORTDiscus and ResearchGate) was conducted up to 28 February 2020. Additionally, the reference lists of the included articles were inspected manually for further unidentified studies. The inclusion criteria were healthy participants of any age, a rating scale used to measure RPE, resistance exercise of any type, one cohort receiving no other intervention, and must present data from one of the following outcome measures: EI, HR, BP, EMG or BLa. Weighted mean effect sizes (*r*) were calculated using a random-effects model. Heterogeneity was assessed using the *τ*^2^ and *I*^2^ statistics. Moderator analysis was conducted using random-effects meta-regression.

**Results:**

One-hundred and eighteen studies were included in the qualitative synthesis, with 75 studies (99 unique cohorts) included in the meta-analysis. The overall weighted mean validity coefficient was large (0.88; 95% CI 0.84–0.91) and between studies heterogeneity was very large (*τ*^2^ = 0.526, *I*^2^ = 96.1%). Studies using greater workload ranges, isometric muscle actions, and those that manipulated workload or repetition time, showed the highest validity coefficients. Conversely, sex, age, training status, RPE scale used, and outcome measure no significant effect.

**Conclusions:**

RPE provides a valid measure of exercise intensity and physiological exertion during resistance exercise, with effect sizes comparable to or greater than those shown during aerobic exercise. Therefore, RPE may provide an easily accessible means of prescribing and monitoring resistance exercise training.

*Trial Registration* The systematic review protocol was registered on the PROSPERO database (CRD42018102640).

**Supplementary Information:**

The online version contains supplementary material available at 10.1186/s40798-021-00386-8.

## Key Points

- Ratings of perceived exertion is a useful and valid measure of resistance exercise intensity.
- Validity coefficients were greater in studies that manipulated workload or repetition time compared to studies that manipulated the number of repetitions or the rest interval time.
- Participant sex, age, training status, and the RPE scale used had no significant effect on RPE validity.

## Background

Ratings of perceived exertion have long been used as a measure of exercise intensity during aerobic exercise [1], with many scales designed and validated for use during exercise of this type. There is a substantial body of evidence to suggest that RPE is a valid measure of exercise intensity and physiological exertion during cardiovascular exercise [2] and team sport training and competition [3]. However, the evidence to support the use of RPE during resistance exercise is less clear and to date no meta-analysis has investigated the validity of RPE during resistance exercise. It has been suggested that it is important to design and validate scales for specific populations, exercise types and modalities [4], and that caution should be taken when using RPE scales with modalities and materials other than those they have been validated for [5]. It has also been proposed that for an RPE scale to be considered a valid measure for use in the clinical and/or health-fitness setting, it must demonstrate both concurrent and construct validity, evidenced by strong positive correlations with physiological variables (e.g., HR) and a previously validated criterion scale, respectively [6]. Despite this, resistance exercise studies and interventions commonly use RPE scales that were not designed or validated for the types of exercise used.

As the use of RPE during resistance exercise has become more widespread, there is a growing body of evidence for the validity of various RPE scales during resistance exercise, including the Borg 6–20 [7], the OMNI-RES [8], and the Borg CR-10 [9] scales. However, due to inherent differences in study design and the unavoidable limitations in every study, validity results from individual studies cannot be taken as a true representation of the validity of RPE [2]. This is highlighted by the wide range of reported validity coefficients within the current literature, with correlation magnitudes reported ranging from *r* = 0.52 [10] to *r* = 0.995 [11] during isometric elbow flexion alone. Therefore, a synthesis of the body of evidence from various forms of resistance exercise is required to provide a true understanding of the use of RPE during this specific type of exercise.

Previous studies have suggested that there are many factors that could affect the validity of RPE during exercise, and therefore could explain some of the heterogeneity in the results from individual studies. During cardiovascular exercise, Chen et al. [2] assessed the effect of several study and RPE characteristics on the strength of the RPE and exercise intensity relationship, including: participant sex, fitness/activity level, RPE scale used, type of exercise (e.g. running, swimming), exercise protocol (e.g. continuous, discontinuous or maximal, submaximal), and RPE mode (i.e. production mode, where the participants are required to manipulate the exercise intensity to achieve a specific RPE score; or estimation mode, where the participant is required to estimate their perceived exertion while working at a predetermined exercise intensity). The findings of the Chen et al. [2] meta-analysis suggested that the highest validity coefficients were achieved when highly fit, male participants, were maximally exerted, during an unusual task, and when a 15-point Borg scale was used (rather than 21-point, 9-point or Category-Ratio Borg scales). These authors [2] reported mean validity coefficients of between *r* = 0.57 and 0.72 depending on the outcome measure were used, and while outcome measure did not have a significant effect on the validity coefficients, there were contradictory findings regarding the effects of moderators depending on which outcome measures used. For example, this study [2] showed that when heart rate (HR), blood lactate (BLa) and VO_2_ were used as outcome measures, RPE in production mode produced significantly higher validity coefficients; however, when ventilation rate was used as the outcome measure, estimation mode produced significantly higher correlations. Likewise, while the highest validity coefficients were obtained from male participants, when BLa was used as the outcome measure, female participants produced significantly higher validity coefficients.

Different experimental designs have produced conflicting findings when using RPE in a resistance exercise setting. Research examining the effect of age on RPE response has suggested that older people require a higher torque to elicit the same RPE score as younger individuals in production mode [12]. Likewise, it has been shown that younger individuals may produce higher RPE scores than older individuals, for the same intensities, during estimation mode tasks [13]. While conversely, other studies have suggested that there is no difference in RPE score due to age [14, 15]. Similar contradictory results are found for the effect of sex, with some studies showing no differences in RPE based on sex [16, 17], while others show females report higher RPE scores during upper and lower body exercise [9].

Ratings of perceived exertion can be accurate in both estimation [18–20], and production mode [13, 20, 21] during resistance exercise, but it is not currently clear whether one produces greater validity coefficients than the other. Additionally, it is possible that upper body exercises may produce higher RPE results than lower limb exercises [9], and that RPE ratings that focus on the specific active muscle group produce higher RPE results than those that take into account overall or whole-body exertion [22]

These large differences in validity coefficients, contradictory findings relating to moderator variables, and the results from previous studies using other forms of exercise (e.g., cardiovascular) confirm the need for quantitative assessment of the validity of RPE during resistance exercise and a greater understanding of which factors, if any, affect the validity of RPE during this type of exercise. This clarity would allow future studies and exercise interventions to use appropriate RPE scales and adapt their protocols to best utilise RPE depending on the exercise type and participant characteristics. Therefore, this study aims to: (1) conduct a systematic review and meta-analysis to collate the current findings and assess the validity of RPE during resistance exercise, and (2) perform moderator analysis to examine which participant, exercise, RPE scale and study design characteristics may affect the validity of RPE during resistance exercise.

## Methods

### Search Strategy

This systematic review was conducted following the Preferred Reporting Items for Systematic Reviews and Meta-Analyses (PRISMA) guidelines. The review protocol was registered with the International Prospective Register of Systematic Reviews (PROSPERO) and was last updated on 15 June 2020 (registration number CRD42018102640).

A systematic computer-based literature search, ending 28/02/2020, was conducted using the following databases and websites: PubMed, Web of Science, SPORT Discus and Research Gate. Three levels of search terms were used; Level 1: RPE OR perceived OR ‘perceived exertion’ OR ‘perceived effort’ OR exertion OR effort OR perception; Level 2: intensity OR ‘exercise intensity’ OR ‘heart rate’ OR HR OR ‘blood pressure’ OR BP OR EMG OR lactate OR workload OR work OR load; and level 3: concentric OR eccentric OR isometric OR resistance OR resistive OR ‘resistance exercise’ OR ‘concentric exercise’ OR ‘eccentric exercise’ OR ‘isometric exercise’. Searches were conducted for level 1 AND level 2 AND/OR 3.

The reference lists of original studies and reviews were also examined to identify any additional articles of interest. Where the researchers were unable to gain access to the full research article, corresponding authors were contacted to ask for a copy of the paper; two full texts were received for evaluation [23, 24]. Where possible, key authors in this field were contacted, to ask for relevant unpublished or in-press data. Additionally, a call for unpublished or in-press data was also placed on Research Gate, which yielded one response [25]. Finally, studies that failed to present the data required for the quantitative analysis, but otherwise met the eligibility criteria (“Eligibility Criteria” section), were sent a request for the missing data; one author replied to this call [26].

Retrieved studies were downloaded to EndNote X8 (Thomson, Reuters, Carlsbad, California, USA) and duplicates were removed. The titles and abstracts of the retrieved studies were screened against the eligibility criteria by two independent reviewers (JL and JS). After this initial assessment, the full texts of papers deemed to meet the eligibility criteria were then assessed using the same criteria, by the same two independent reviewers. Any conflicts were resolved by a third reviewer (JW).

### Eligibility Criteria

The eligibility criteria for inclusion in qualitative synthesis were: (1) Only original research articles were included. (2) Studies must use at least one group of healthy participants. ‘Healthy’ was defined as having no injury or illness that could affect the participant’s performance, having no clinical diagnosis of any condition or dysfunction, and were not taking any medication that could affect exercise performance or cardiovascular function; there were no age restrictions on the participants used. (3) Studies must have used a resistance exercise modality, defined as a systematic series of exercises that cause muscles to work or hold against an applied force or weight [27]; dynamic, eccentric only, concentric only, isometric, and isokinetic exercises were all acceptable. (4) Data must be presented for at least one group that did not receive any confounding interventions e.g., supplementation. (5) A rating scale must have been used to measure perceived exercise intensity, exertion, or discomfort. (6) Only studies written in English could be accepted. There were no restrictions on publication date, and un-published or ‘grey’ literature, for example theses and conference proceedings were accepted.

**Fig. 1:**
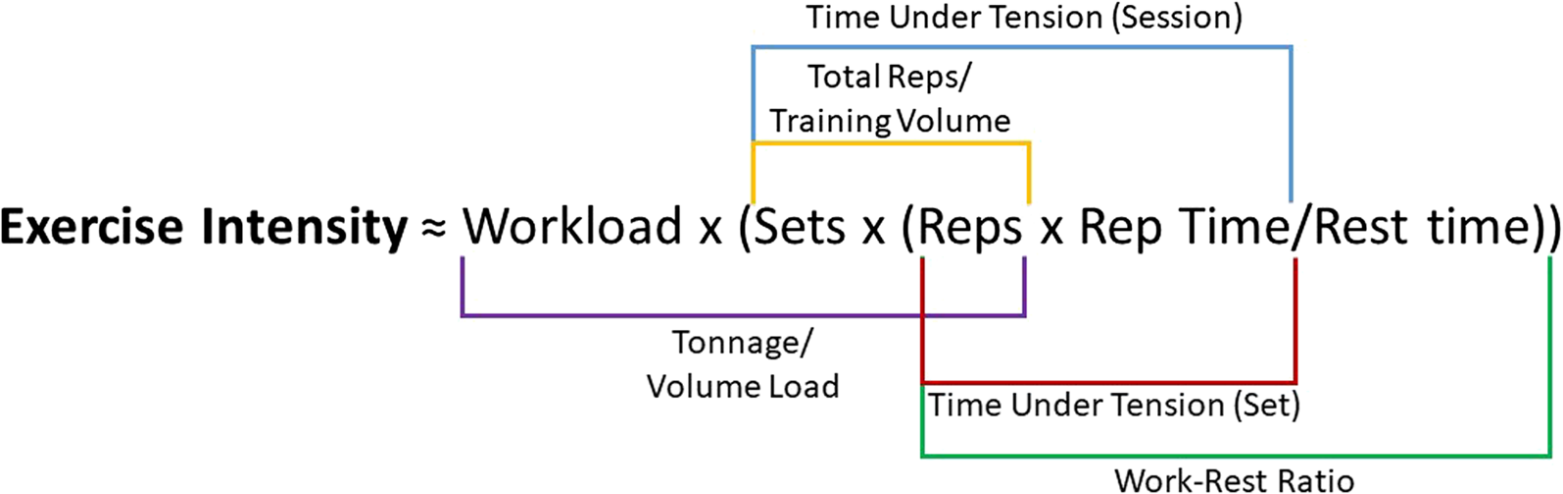
Exercise intensity variables and common terminology. Rep(s) = repetition(s), Workload could be substituted with force, torque or %MVC

For inclusion in the quantitative (meta) analysis, all of the qualitative synthesis criteria must have been met and then additionally: (7) Studies must have presented one of the following outcome measures: exercise intensity (EI), HR, BP, EMG or BLa. In this study, EI is defined as the interaction of workload, number of sets, number of repetitions, repetition time and rest time (Fig. 1); thus, EI can be modified by changing one or more of these variables. (8) If using a direct measure of EI, there must have been an objective change between trials/conditions; for example, studies that increased load and decreased repetitions to match tonnage/volume load between conditions, were not included in the quantitative analysis. (9) Data must have been presented in one of the following forms for RPE and at least one of the physiological exertion measures and/or EI: correlation or linear regression (*r* or *r*^2^ values) or means and standard deviation from two or more trials/conditions (e.g., time points or workloads).

### Data Extraction and Coding

Study eligibility assessment, risk of bias rating and data extraction/coding were conducted independently by two reviewers (JL and JS). All data were extracted and coded onto a custom Excel spreadsheet. Studies in the meta-analysis were coded for participant, exercise, RPE scale and study features (Table 1) to allow for meta-regression analysis of possible moderators. The ‘muscle action’ used in each study was coded for studies using dynamic (i.e. a concentric followed by an eccentric contraction), concentric only, eccentric only, or isometric. The part of the body used in the exercise, or ‘body segment’ was also coded, i.e., an upper body, lower body, or whole-body exercise. Continuous and intermittent exercise ‘protocols’ were included and coded (e.g., an incremental test vs. a traditional weight training session, respectively). Where a study actively adjusted workload between trials or conditions, the workload range (maximum workload − minimum workload) was also coded as percentage of one-repetition maximum (%1RM) or percentage of maximal voluntary contraction (MVC). Ratings of perceived exertion scale properties were recorded including: scale used, number of points on scale (e.g. the Borg 6–20 scale is a 15-point scale); fixed maximum, whether the scale has a fixed or open maximum (e.g. maximum = 10 or an open ended scale like the CR-10); rating mode (estimation or production); rating type, i.e. rating exertion in the active muscles only (RPE-AM), overall body (RPE-O), or whole session (S-RPE). Finally, if EI was manipulated, the variables used to do so were coded.

**Table 1:** Participant and study features and coding

Type	Feature	Categories	Coding
Participant	Age of participants	Mean years	Nos.
	Sex of participants	Male	1
		Female	2
		Both	3
	Resistance training level	Sedentary	1
		< 6 month	2
		> 6 month	3
		> 1 Year	4
		Elite level	5
Exercise	Muscle action	Dynamic	1
		Concentric	2
		Eccentric	3
		Isometric	4
	Body segment	Upper	1
		Lower	2
		Whole	3
	Protocol	Continuous	1
		Intermittent	2
	Workload range	(% 1RM)	Nos.
RPE Scale	Scale used	Borg 6–20	1
		CR-10	2
		OMNI-RES	3
		ERF	4
		Borg words	5
		IES	6
		NRS	7
		PTD	8
		RES + RIR	9
	Number of points	–	Nos.
	Fixed maximum	Yes	1
		No	2
	Rating mode	Estimation	1
		Production	2
	Rating type	Active muscle	1
		Overall	2
		Sessional	3
Study	Outcome measure	EI	1
		HR	3
		EMG	3
		BLa	4
	EI variable manipulated	Workload	1
		No. reps	2
		Rep time	3
		Rest time	4

Coding, nominal coding used to allow analysis as a categorical variable. ERF, estimated repetitions to failure [28], Borg words, Borg CR-10 verbal cues with no numerical cues [29]. IES, Isometric Exercise Scale [30–32], NRS, Numerical Rating Scale [11, 18], PTD, perceived task duration [33], RES + RIR, resistance exercise specific RPE with repetitions in reserve [19]

If a study did not report a variable or their result did not fit into one of the pre-defined categories, a code of ‘99’ was given and the study was excluded from the meta-regression analysis for that variable. Negative correlation *r *values for repetition velocity and RPE or knee joint angle and RPE, that represent increases in time under tension and workload, respectively, were included as positive values.

Information from studies fulfilling the qualitative inclusion criteria, but not the quantitative, were synthesised using a narrative/thematic summary method.

### Risk of Bias in Individual Studies

The risk of individual study bias in methodology or reporting was assessed, independently by JL and JS, using a 9-point scale designed in-house for RPE validity studies (see Additional file 1: Table S1). The 9 criteria assessed were: (1) participant eligibility criteria specified and fulfilled, (2) participant information given (must include: age, sex and training status), (3) a priori power analysis/sample size calculation completed, (4) exercise type (dynamic, isometric etc.) and movement (squat, bench press etc.) specified, (5) exercise intensity specified (including load, number of sets, number of repetitions, repetition time and rest interval time), (6) exact RPE scale used (including any modifications), (7) RPE instructions are specified, (8) anchoring procedures are specified, (9) a measure of repeatability/reliability was reported. Each criterion was given a score of 0 (indicating the criteria was not fulfilled or was not reported) or 1 (indicating the criteria was fulfilled and reported). A score of 0–3 was considered ‘high risk’, 4–6 was considered ‘moderate risk’, and 7–9 was considered to have a ‘low risk’ of bias.

### Data Analysis

#### Publication Bias

To examine the possibility of publication bias in this body of evidence, funnel plots of individual Fisher z values versus their corresponding standard errors were manually examined for signs of asymmetry (Additional file 1: Fig. S1). Duval and Tweedie’s trim and fill method was then used to look for missing studies and adjust the point estimate accordingly. Following this, the Classic fail-safe N was calculated to elucidate the number of unpublished non-significant studies that would be needed to make the result of this analysis non-statistically significant (*p* < 0.05). Finally, Orwin’s fail-safe N was calculated to examine how many unpublished studies would be required to reduce the calculated point estimate to a ‘medium’ or ‘low’ effect size (*r* < 0.5 and *r* < 0.3 respectively).

#### Synthesis of Results

All analyses were conducted using Comprehensive Meta-Analysis software (version 3, Biostat Inc., Englewood, NJ, USA). Some of the eligible studies reported multiple outcome variables for the same participants. Therefore, 2 separate random-effects meta-analyses were conducted; ‘all measures’, which included any outcome variable, and ‘EMG’ as the only outcome measure. All studies/cohorts reporting EI as the outcome measure were included in the main ‘all measures’ analysis therefore a separate analysis was not required. There were insufficient studies to conduct separate analyses for HR, BP or BLa, thus studies reporting these variables were only included in the ‘all measures’ analysis. For each analysis the mean sample size weighted correlation coefficient (*r*), 95% confidence interval (CI), 95% prediction interval (PI), and significance level (*p*) were calculated. Between-study heterogeneity was assessed using standard Chi Squared test (Cochran’s test), *τ*^2^ and *I*^2^ statistics.

#### Sensitivity Analysis

Sensitivity analysis was conducted on each meta-analysis by systematically removing one study from the analysis to assess the effect on the point estimate. As no single study significantly affected the point estimate, all of the studies eligible for each analysis were included.

#### Moderator Analysis

Where statistically significant between-estimate heterogeneity was shown by the Chi Squared test (*p* < 0.01), meta-regression analysis was conducted to determine the effect of participant and study characteristics on the effect sizes reported. All moderators were assessed separately, using univariate regression analysis, and then used in combination to find the most effective multivariate regression model. Individual moderators and models were assessed using the *τ*^2^ (unadjusted *τ*^2^ vs adjusted *τ*^2^) and *R*^2^ statistics.

## Results

### Literature Search

**Fig. 2:**
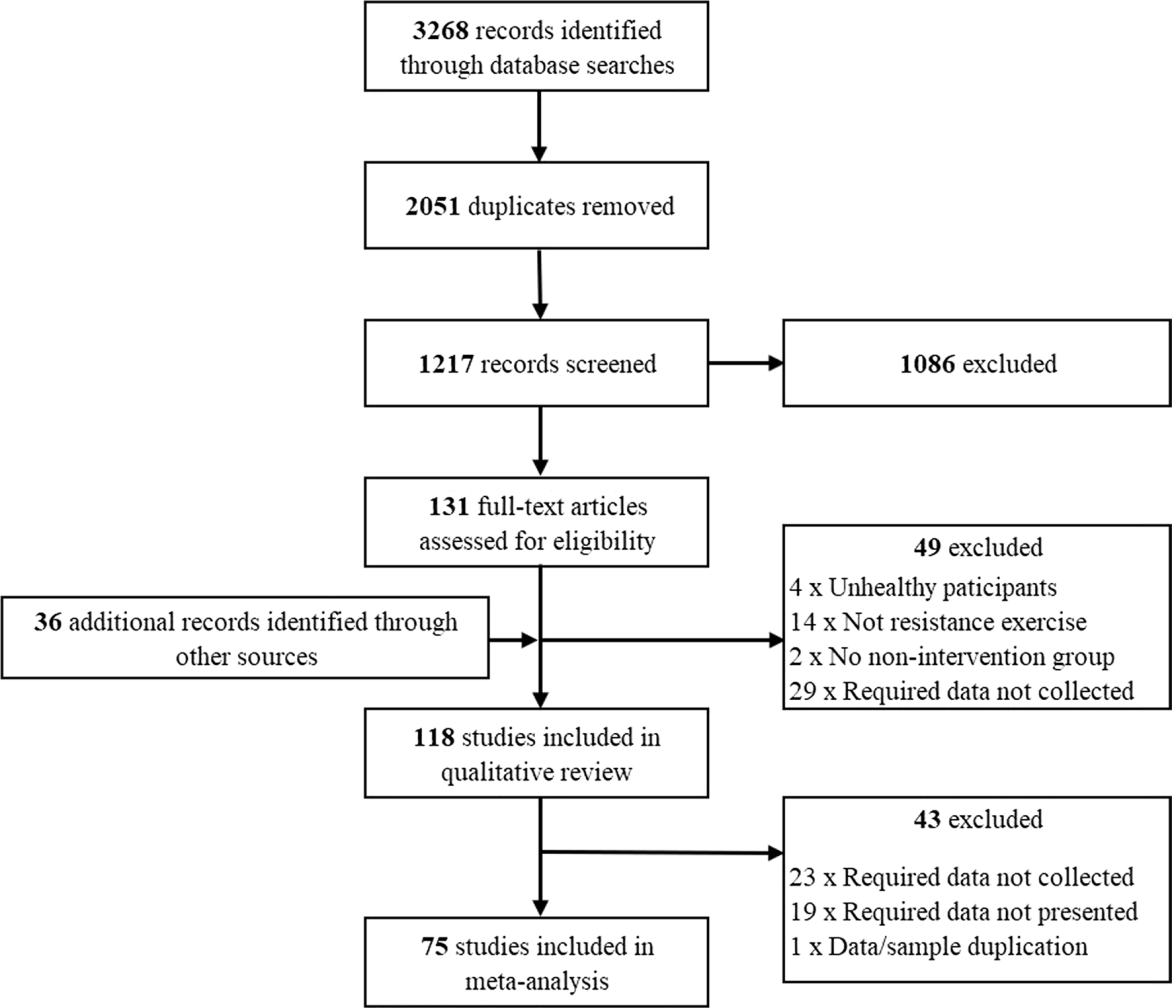
PRISMA flowchart illustrating the phases of the search and study selection

As seen in Fig. 2, the primary searches revealed 3268 potentially relevant studies. After removing 2051 duplicates, the titles and abstracts of 1217 studies were examined against the inclusion criteria. Of the 1217 studies, 131 appeared to adhere to the inclusion criteria and as such the full texts were then reviewed. During full text review the reference lists of each article were examined for additional articles; 36 additional articles were identified, and these full texts were also examined. One hundred eighteen studies were eligible for inclusion in the qualitative analysis (49 excluded), with 75 studies included in the final quantitative analysis (Fig. 2).

#### Study Characteristics

Of the 75 studies eligible for the quantitative analysis, the overall risk of bias was ‘low’ in 44 studies and ‘medium’ in 31 studies. No studies included showed a ‘high’ risk of bias. Only 13 studies included/reported a measure of inter-session reliability. The primary analysis (all measures) included 75 studies [4, 5, 7–13, 15, 18, 19, 21, 22, 25, 26, 28–85], with 99 unique cohorts (measures: EI = 89, HR = 2, EMG = 6, BLa = 2). These 99 cohorts contained a total of 2231 participants. The secondary analysis (EMG only) used 7 studies [11, 12, 41, 52, 53, 63, 82], containing 8 unique cohorts with a total of 340 participants.

### Publication Bias

There was some evidence of asymmetry in the funnel plot for the primary analysis; however, Duval and Tweedie’s trim and fill method did not add or remove any studies and made no adjustment to the point estimate. Additionally, the Classic fail-safe N revealed that 158,597 non-significant studies would be required to render the analysis non-significant (*p* > 0.05). Likewise, the Orwin’s fail-safe N analysis showed that 108 and 268 studies, each with a correlation of *r* = 0.00, would be required to reduce the weighted mean effect size to medium (*r* < 0.5) and small (*r* < 0.3) respectively.

### Primary Analysis: Validity of RPE Using All Outcome Measures

**Fig. 3:**
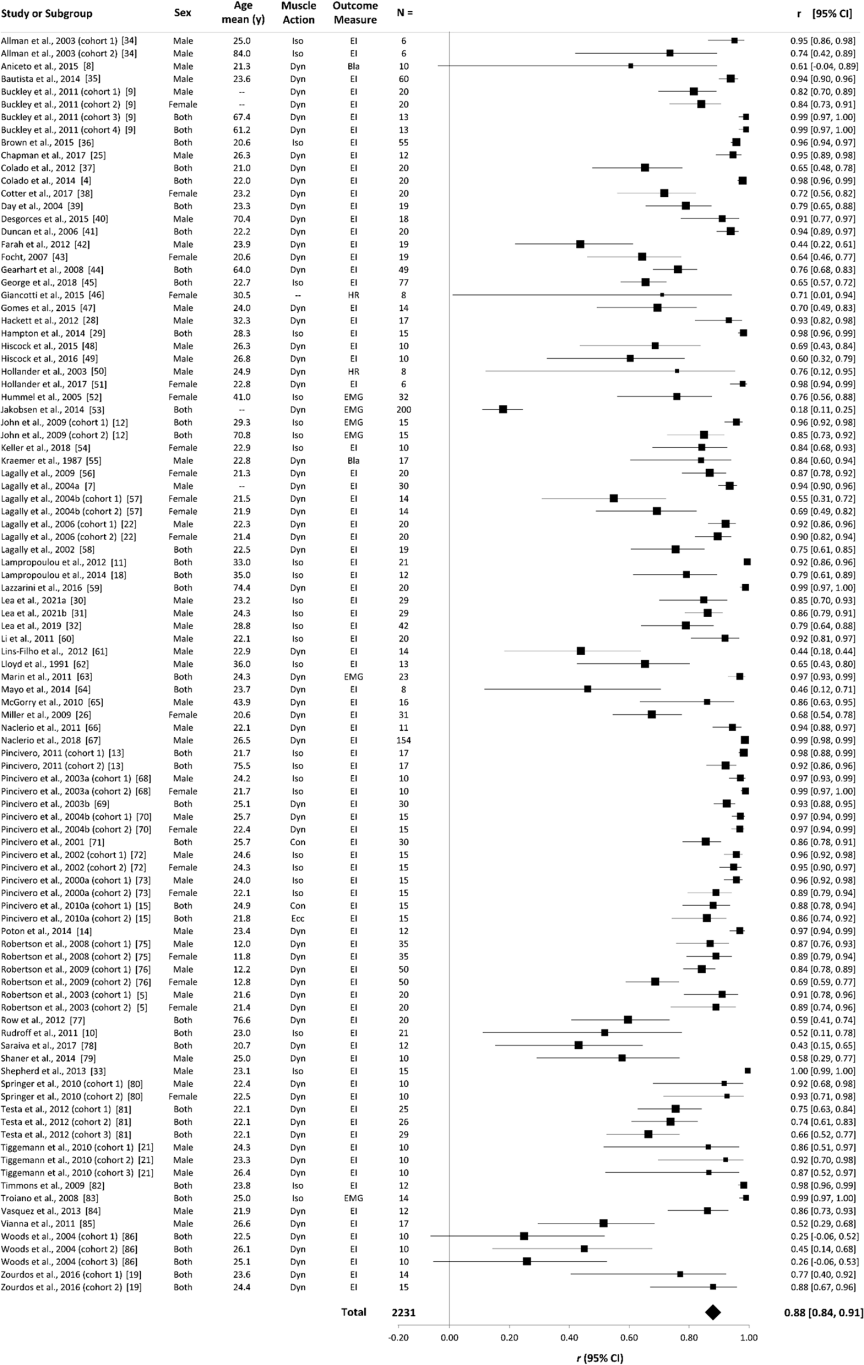
Forest plot showing the weighted validity coefficients (solid squares) and 95% confidence intervals (solid horizontal lines) for each study included in the ‘all measures’ analysis. The bottom row indicates the overall random-effects validity coefficient (solid diamond). Key study characteristics are presented next to each study; the ‘–' symbol indicates data that were unavailable

Figure 3 shows the validity coefficients and 95% confidence intervals for each of the studies, and the weighted mean effect size for the relationship between RPE and the measure of EI or physiological exertion. The overall weighted mean validity coefficient was very large, *r* = 0.88 (95% CI 0.84–0.91; 95% PI − 0.07 to 0.99; *p* < 0.001). There was significant between study heterogeneity (*p* < 0.001); total between study variance was *τ*^2^ = 0.526, with a high level of true/explainable between study heterogeneity (*I*^2^ = 96.1%).

### Secondary Analysis: Validity of RPE with EMG as the Outcome Measure

**Fig. 4:**
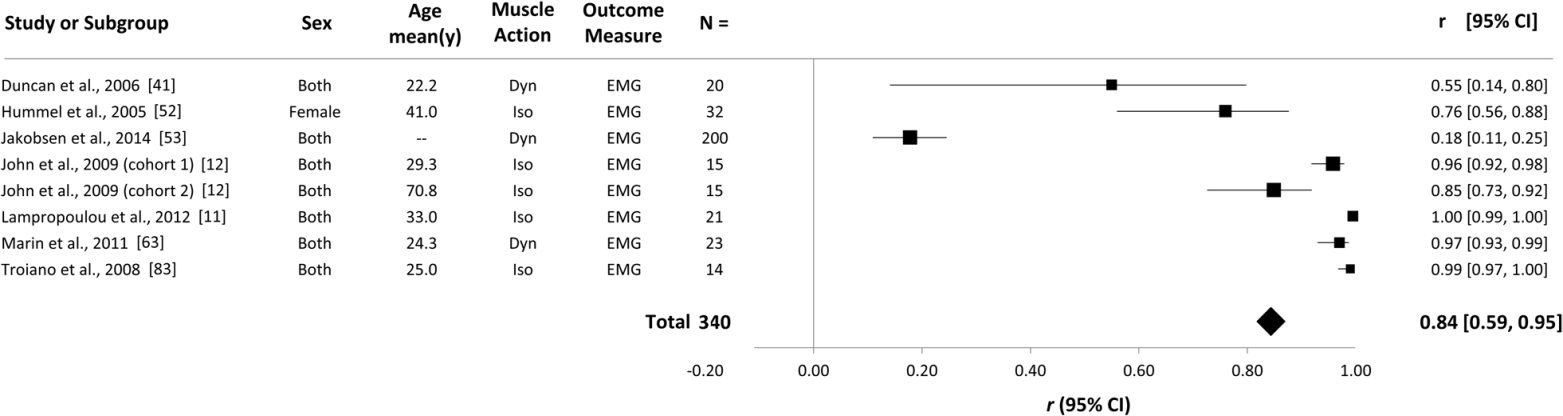
Forest plot showing the weighted validity coefficients (solid squares) and 95% confidence intervals (solid horizontal lines) for each study included in the ‘EMG’ analysis. The bottom row indicates the overall random-effects validity coefficient (solid diamond). Key study characteristics are presented next to each study; the ‘–' symbol indicates data that were unavailable

As shown in Fig. 4, the weighted mean effect size for the 8 cohorts reporting EMG as the outcome measure was also very large, *r* = 0.84 (95% CI 0.56–0.95; 95% PI − 0.68 to 1.00; *p* < 0.001). As with the primary analysis, there was significant between study heterogeneity (*p* < 0.001), between study variance was *τ*^2^ = 0.624, and the level of true between study heterogeneity was high (*I*^2^ = 97.3%).

### Moderator Variables and Meta-Regression

As a significant level of explainable between study heterogeneity was present, meta-regression was used to examine which participant, exercise, scale, and study characteristics may affect the validity of RPE during resistance exercise. The secondary analysis contained data from 8 cohorts, 6 of which were included in the ‘all measures’ analysis. Additionally, the validity coefficients, variance and heterogeneity were comparable; therefore, moderator analysis was only conducted on the primary analysis (all measures).

**Fig. 5:**
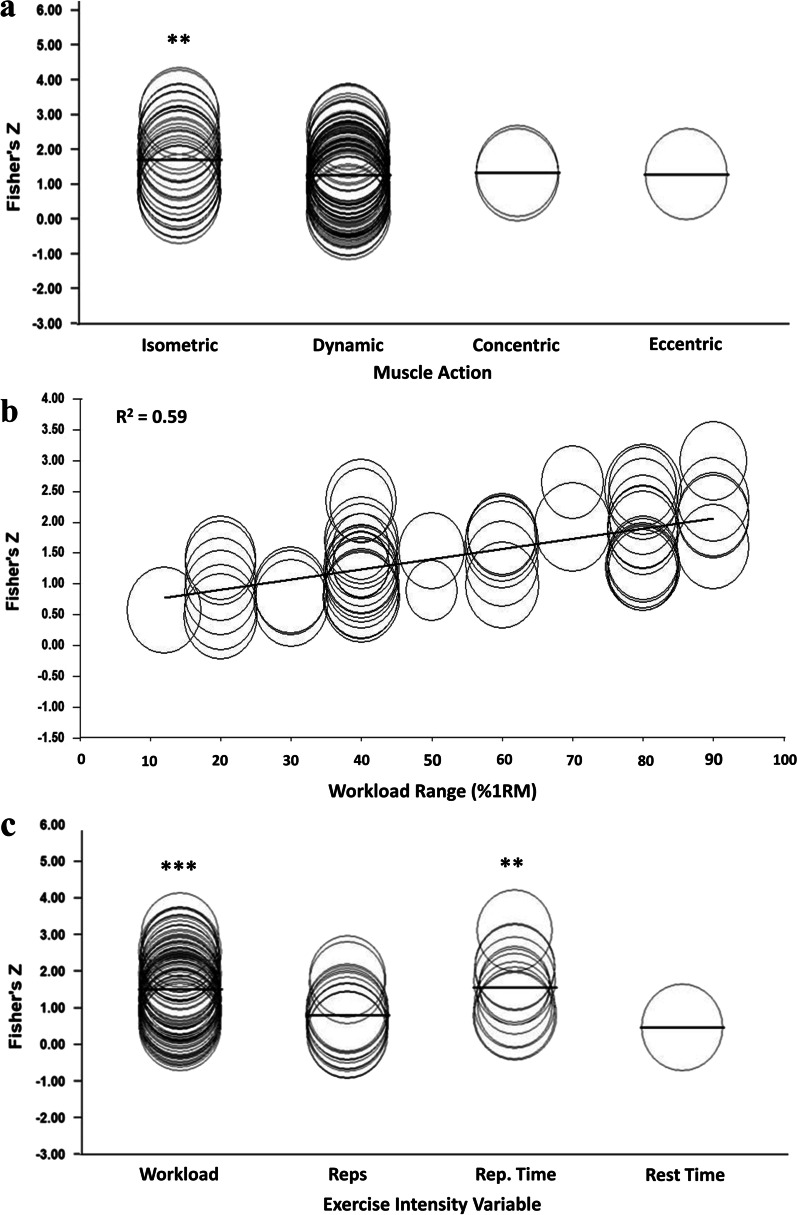
Significant results of the random-effects univariate meta-regression analyses. **a** The effect of muscle action, **b** the effect of workload range, and **c** the effect of exercise intensity variable on Fisher’s Z transformed validity coefficients. **p < 0.01; ***p < 0.001

Univariate regression analysis showed no statistically significant moderating effect of the participant characteristics: age, sex or resistance training level (*p* < 0.05). Likewise, the exercise characteristics: body segment and protocol; scale characteristics: scale used, number of points, fixed maximum, rating mode and rating type; and the study characteristics: outcome measure and risk of bias, had no effect on the reported validity coefficients (*p* < 0.05). Conversely, univariate analysis of 98 cohorts showed that muscle action did significantly affect the validity coefficient, with isometric exercise giving significantly (*p* = 0.004) higher values than dynamic, concentric, or eccentric contractions (Fig. 5a). Likewise, analysis of the 56 cohorts that reported a quantifiable change in workload, showed that the workload range significantly (*p* < 0.001) affected the validity coefficients with studies that used greater ranges showing larger effect sizes (Fig. 5b). The EI or physiological exertion measure used had no effect on the validity of RPE; however, for the cohorts using EI as the outcome measure (*n* = 83), manipulation of workload and repetition time showed significantly higher effect sizes (*p* < 0.001 and *p* = 0.002 respectively) than manipulation of the number of repetitions or the rest interval time (Fig. 5c).

Various multivariate regression models were built using the coded characteristics; workload range and EI variable showed collinearity and so could not be included in the same multiple regression models. The strongest model included: sex, rating type, and workload range. There were 50 unique cohorts that reported data for all 3 of these variables. This model explained 64% of the between study heterogeneity (*R*^2^ = 0.64), reducing the total between study variance from *τ*^2^ = 0.391–0.142. There was still a significant amount of variance not explained by the model (*p* < 0.001), and the amount of explainable variance was still high (*I*^2^ = 86.2%). The meta-regression equations for both the univariate and multivariate analyses are shown in Additional file 1: Table S2.

## Discussion

### RPE During Resistance Exercise

This is the first systematic review and meta-analysis to assess the validity of RPE as a measure of exercise intensity during resistance exercise modalities. This study aimed to inform researchers, clinicians, athletes and coaches, so that RPE can be used more effectively in studies and interventions, by: (1) assessing the validity of RPE during resistance exercise, and (2) examining which participant, exercise, scale and study characteristics may affect the validity of RPE during this type of exercise. The results of this study demonstrate that RPE is a valid measure of resistance exercise intensity, with strong correlations to EI, HR, EMG, and BLa giving a weighted mean validity coefficient of *r* = 0.88; therefore, RPE validity may be higher during resistance exercise than was previous shown for aerobic exercise [2] and during team sports [3]. The use of RPE in aerobic exercise is widespread, used by exercise professionals and recreational athletes alike; however, the use of RPE, especially amongst recreational athletes, in resistance exercise is not yet as common. Our results suggest that this is a tool that is both accurate and effective in the resistance exercise setting, and as such could be of great benefit if used more widely to optimise programming.

### Participant Characteristics

#### Age

The results of the meta-regression analysis showed that the age of participants did not statistically significantly affect the validity of RPE (*p* < 0.05). While results from different age groups may show statistically similar effect sizes (validity coefficients), this does not discount the possibility that there are consistent differences in the absolute magnitude of the responses, between age groups. There are conflicting results within the current literature around possible age differences in RPE responses. It has been suggested that at sub-maximal levels of exertion, older adults report lower RPE values than younger adults [13, 34]; with no difference shown at maximal fatigue [34]. Likewise, in production mode, older participants have been shown to produce significantly higher %MVC contractions at set submaximal RPE levels [12, 13]. Conversely, no significant differences in RPE response were shown between young and older adults during maximal or submaximal isometric back extension [86], submaximal isometric arm abduction [87] or submaximal hand grip and leg extension exercises [88]. It should be noted that all the above studies used isometric exercise and with the possible exception of back extension [86], all used single joint exercises. One study [9] that used a mixture of single and multi-joint, upper and lower body dynamic exercises, also showed conflicting results. This study concluded that on the whole, results from the CR-10 scale showed no differences between age groups, especially during leg muscle training. However, age differences were seen during single-joint arm exercises and low-intensity leg extensions.

The results of our analysis show that RPE provides a valid representation of EI irrespective of age. The mixed results in the literature make it unclear whether there is a reliable difference in RPE ratings caused by age. If there is an age dependent difference, it would seem likely that older participants give lower RPE ratings at the same relative workload or older participants will produce higher relative loads at a set RPE, and this difference is likely to be more pronounced during single joint upper body exercise. More research is required to examine whether there is an age dependent difference, and to explore the factors that may cause this difference to be present, to increase or decrease.

#### Sex

Univariate moderator analysis showed that sex did not have a statistically significant effect on the validity of RPE results, and while the multivariate model explaining the largest amount of heterogeneity did contain sex as a variable, the amount of variance explained by sex was still not statistically significant (*p* < 0.05). These results suggest that there is no sex dependent difference in RPE validity. This result is contrary to the main findings of Chen et al. [2], who showed higher validity coeffects in males, with all outcome variables except BLa where female validity was higher. It should be noted that in Chen’s study, there were fewer females in the analysis of each outcome variable; and for BLa, females made up only approximately 25% of the total sample; this together with the conflicting results between outcome measures, highlights the need for further investigation with greater numbers of studies using female participants.

Two studies [82, 89] in the current analysis suggested that males give significantly higher RPE ratings, at the same relative exercise intensities than females. These studies used two novel exercise modalities, eccentric elbow flexor exercise [89] and upper trapezius shoulder elevation exercise [82], which raises the question as to whether certain types of resistance exercises do elicit a sex difference in RPE response. However, the vast majority of the current RPE research suggests there are no sex moderated differences; including during: 1RM prediction [16], concentric contractions [15, 71], eccentric contractions [15], isometric contractions [68, 73], and dynamic resistance exercise [90]. Likewise, RPE ratings for active muscle and overall body [22], in estimation mode [9, 69, 79], in production mode [72], and in estimation mode in older adults [17, 44] have all shown no sex differences. Therefore, the weight of the available evidence suggests that there is no significant difference in mean RPE responses between males and females, and that individual differences are likely to have a far greater effect than any sex related differences. Additionally, our meta-regression analysis revealed that RPE was an equally valid measure of EI in males and females.

#### Resistance Training Level

Moderator analysis showed that the resistance training level of the participants had no statistically significant effect on the validity of the RPE response given. There are a limited number of studies that have directly compared groups with different resistance training experience. Comparisons of trained and untrained participants during back squat, bench press and arm curl exercises showed no differences in RPE [91]. Likewise, no differences were seen between novice and recreationally trained weightlifters during bench press exercises at the same relative intensities [57]. Conversely, it has been suggested that, at low relative training volumes, novice athletes are less accurate at representing actual training load, giving lower RPE ratings than well-trained participants [80]. Likewise, in estimation mode, novice squatters gave lower RPE scores than experienced squatters at maximal load [19]. However, it was suggested that this could be due to the inability of novice squatters to perform a true 1RM test in dynamic squatting. In production mode, sedentary individuals produced significantly lower relative forces than strength trained individuals, at both low and high submaximal RPE scores [21]. This result would seem to contradict the findings of the previous two studies, as this would suggest that novice athletes would perceive relative loads as harder than trained participants; but comparison of estimation and production mode in this way may not be valid.

Several studies have suggested that RPE ratings significantly decreased in participants following a training programme using the same exercise [40, 44, 92–94]. However, all these studies compared RPE at set absolute loads or at relative loads based on 1RM tests performed before the training programme was completed. Additionally, these studies all showed an increase in 1RM following the training; therefore, the reductions in RPE are merely representative of an increase in strength and thus, the absolute loads becoming lower relative loads. Based on this, Gearhart et al. [95] concluded that as relative load and RPE decrease concurrently, RPE can be used to track strength training in older individuals. One study was identified that compared RPE at relative workloads based on pre- and post-intervention 1RM [44]. This study showed that in production mode, both the absolute and relative loads lifted increased, at RPE scores of 4, 6 and 8. This result would support the findings of Tiggemann et al. [21], that trained individuals may produce higher relative forces at set RPE levels than novices.

Based on the current available evidence, including our findings, there are no clear differences in RPE ratings caused by training level and experience and RPE is equally valid across the difference experience levels. However, it is possible that in production mode trained individuals will work at higher relative loads than novices.

### Exercise Characteristics

#### Body Segment

The body segment used had no statistically significant effect on the validity of the RPE responses given. As previously stated, some research has suggested that lower-body exercises give more consistent inter and intra-scale RPE results, than upper body exercises [9]. In estimation mode, it has been suggested that lower body exercise elicits higher RPE results than upper body, possibly due to the larger muscle mass involved [64]. Likewise, in production mode, at lower RPE values, higher relative loads were produced during bench pressing than during leg pressing; although, this difference was not present at higher sub-maximal RPE values [21]. In contrast to this, no significant differences where shown, between upper and lower body exercise, when using RPE to estimate number of repetitions until failure [28] or to predicted maximal load [16]. However, these two analyses are dealing with validity rather than differences in actual ratings given. Therefore, RPE is equally valid for upper, lower or whole-body exercise; while there is limited evidence that the larger muscle mass of the lower body will give higher RPE estimations or lower relative loads in production mode.

#### Protocol

Chen et al. [2] showed that during aerobic exercise, using HR as the outcome measure, random intermittent exercise protocols produced significantly lower validity coefficients than progressive continuous, progressive intermittent and submaximal protocols. While no individual study included in our review directly compared difference protocol types, moderator analysis of 92 cohorts, containing each of these study protocols, showed no statistically significant differences in RPE validity during resistance exercise.

#### Workload Range

Workload range was the biggest single predictor of between study variance, with univariate analysis showing that workload range explained 59% of the heterogeneity. Workload range was also the only significant variable in the multivariate model that explained 64% of the between study variation. The meta-regression showed that greater workload ranges led to significantly greater effect sizes (*p* < 0.001). This is to be expected, as a study that has compared RPE results at, for example, 40% and 50% of 1RM (a 10% range) will show a smaller effect size than one that has used 10% and 90% of 1RM (an 80% range). This variable could only be coded for studies that actively manipulated workload; therefore, any studies that adjusted repetition time, number of repetitions or sets, or rest time could not be included. As a result, only 56 cohorts were included in the univariate analysis and 50 cohorts in the multivariate analysis. It is likely that a far larger amount of the total between study variance would have been explained if exercise intensity, calculated using all 5 of the above variables, could have been quantified for each study.

#### Muscle Action

There were no statistically significant differences in validity between dynamic, concentric, or eccentric contractions (*p* < 0.05); whereas isometric contractions showed significantly (*p* = 0.004) higher validity coefficients than the other contraction types. It is possible that, due to the elongated nature of most isometric contractions, this increase in validity is linked in some way to the increased validity shown in studies that manipulated repetition time, when compared to number of repetitions or rest interval time. However, as none of the included studies directly compared isometric contractions to any other type, further investigation is required to explain the underlying mechanisms behind these differences.

Previous research has demonstrated that both RPE and perceived pain values are significantly lower during eccentric contractions than during concentric [26, 50] and dynamic [26] contractions at the same absolute loads. This difference seems to be due to the increased 1RM capacity during eccentric contractions when compared to concentric. Indeed, RPE was consistent between eccentric and concentric contractions when each contraction was conducted at the same relative load, based on the 1RM for each contraction type [96]; with eccentric loads approximately 20% higher than concentric loads at the same RPE levels [97].

### Scale Characteristics

#### Scale Used, Points and Fixed Maximum

Nine different RPE scales were used in the studies included in this review, including Borg’s 6–20, Borg’s CR-10, the OMNI-RES, Estimated-Repetitions-to-Failure (ERF) [28], Borg’s scale verbal cues only [29], Isometric Exercise Scale (IES) [30–32], Numerical Rating Scale [11, 18], Perceived Task Duration (PTD) [33], and Resistance Exercise Specific Scale with Repetitions in Reserve [19]. The specific RPE scale used did not influence the reported effect sizes. Likewise, differences in the RPE scale properties, number of points and fixed maximum, had no effect on the validity of RPE.

Ratings of perceived discomfort or pain (RPD) is often used as an analogue of RPE to monitor EI. Indeed, the CR-10 scale was designed for use as a muscular pain scale and later validated as an exertion scale [1]. Comparison of perceived discomfort and perceived exertion has yielded similarly high correlation coefficients (*r* = 0.71–0.86) [50], suggesting that both RPD and RPE are valid metrics that can be used to monitor EI. However, it has been suggested that RPE ratings are higher at a set intensity than RPD [50, 98].

It is common within RPE research to use correlation with a previously validated RPE scale to show the validity of a new scale to measure the construct of perceived exertion; for example, Lagally and Robertson [22] compared the OMNI-RES and Borg 6–20 scales (*r* = 0.94–0.97), Hackett et al. [28] compared ERF and OMNI-RES (*r* = 0.96), Shepherd et al. [33] compared the CR-10 and PTD (*r* = 99), and Lea et al. [30] compared IES and CR-10 (*r* = 0.97). These results, in addition to providing the intended construct validity, further support the findings of our analysis that many RPE scales, despite having different designs, properties, and intended uses can be used interchangeably without affecting the validity of the results.

#### Mode

The results of our meta-regression revealed that there were no statistically significant differences in validity coefficients between estimation and production modes (*p* > 0.05). During aerobic exercise, Chen et al. [2] showed higher validity coefficients for production mode than estimation mode for all outcome measures except ventilation, which had a low number of production studies. This difference may be due to the higher validity coefficients seen in the current analysis for estimation mode during resistance exercise, where it is possible that changes in intensity are more noticeable and quantifiable.

Pincivero, 2011 [13] suggested that when compared to estimation mode, both older and younger adults significantly underproduce isometric leg extension torques at higher RPE levels. However, Morrin et al. [20] showed no significant differences in the %MVC achieved between estimation and production trials during isometric hand grip exercise. These conflicting results prompt further investigation to explore possible differences.

Hampton et al. [29] showed that production mode could be used to produce distinct levels of force at 5 different RPE levels. Morrin et al. [20] suggested that production RPE was sufficiently accurate to self-regulate isometric hand grip training for reducing BP. Likewise, OMNI-RES ratings of 3, 6 and 9 have been show to accurately and reliably produce intensities that are appropriate for improving muscular fitness [56]. A 12-week training intervention, at an RPE of 4 on the OMNI-RES scale, was sufficient to increase post training 1RM in 7 different exercises [17]. Similarly, a 12-week training intervention, prescribed at RPE ratings from 13 to 18 on the Borg 6–20 scale, produced increases in maximal leg press (58%), knee extension strength (20%), and knee extension power (27%) [99]. These results suggest that RPE can be used in both production mode and estimation mode as a valid and accessible means of prescribing and monitoring EI, respectively.

#### Rating Type

This study demonstrated that differences in the rating type, i.e., RPE-AM (active muscles), RPE-O (overall body) or S-RPE (sessional), did not significantly influence the strength of the validity coefficients obtained, suggesting that all three of these methods can be used to monitor EI. Results consistently show that RPE-AM ratings are higher than RPE-O ratings [4, 5, 22, 37, 41, 49, 58, 100], with increasing divergence in these ratings as intensity increases [57, 101]. Ribeiro et al. [102] showed no significant difference between RPE-AM and RPE-O ratings, during circuit weight training; however, this study assessed both RPE types 10, 20 and 30 min after the exercise had finished, when they would normally be assessed immediately following a repetition or set, which could account for these conflicting results.

There are conflicting results in the current literature regarding the outcome of S-RPE compared to RPE-O. It has been suggested that mean RPE-O, taken immediately after each set, elicits significantly higher ratings than S-RPE [103, 104]. Conversely, Day et al. [39] showed non-significant differences in RPE-O (taken immediately post set) and S-RPE (taken 30 min post exercise) at 50% and 70% 1RM, and no differences at 90% 1RM. Likewise, Costa et al. [105] showed no difference between RPE-O, collected immediately after the last set, and S-RPE collected 30 min post exercise. In addition to these results, there are conflicting results concerning the implementation of S-RPE; Kraft et al. [106] suggests there is no difference between S-RPE taken at 10- and 30-min post exercise, while Singh et al. [103] showed significant differences between S-RPE taken at 5- and 10-min when compared to 30-min. Singh suggests that 30-min S-RPE is a better overall indicator of training session intensity.

The optimum implementation of S-RPE requires further investigation, but the results of our analysis would suggest that all three types of RPE rating can all be used to accurately measure EI.

#### Anchoring Procedure

Anchoring is regularly used as part of the standardised instructions given to users to explain how to use the given RPE scale properly. Anchoring aims to give the user a clear understanding of what one or more points on the scale mean in relation to EI. This is often done by anchoring the extremities of the scale, so that the user can then estimate what the other points should feel like based on those anchors. It is suggested that providing standardised instructions and anchoring is important to accurately gauge EI [107].

There are several methods of anchoring, including: memory anchors, exercise anchors and combined memory-exercise anchors. Memory anchors call upon previous experience, for example, maximal is the hardest exertion previously experienced [48–50]. Exercise anchors utilise exercise at a set percentage of the user’s maximum, followed by an explanation of what level on the RPE scale that exercise is; for example, isometric holds at 10% and 100% of MVC for 5 s are 1 and 10 in the CR-10 respectively [108], or following a 1RM lift the participant is told that that is ‘maximal exertion’ on the scale [107]. Finally, the combined memory-exercise anchor uses both methods, anchoring some points on the scale using exercise and others using the participant's memories and estimations [22, 97, 109]. Legally et al. [7] compared these anchoring methods at 6 intensities from 40 to 90% MVC; their results showed no significant differences in the mean rating at each intensity between anchoring groups, however, the exercise and memory-exercise groups did show better reliability between the first two sessions, when compared to the memory group. It would seem that valid results can be obtained using all three anchoring methods. While reliability was suggested to be improved across the first two sessions with the inclusion of an exercise anchor [7], it is likely that this difference will diminish in later sessions due to familiarity with the specific exercise. It should also be noted that no studies were identified that compared any anchoring procedures with a non-anchored group, it may therefore be an assumption that anchoring is important to increase validity and/or reliability.

### Study Characteristics

#### Outcome Measure

Moderator analysis showed that RPE validity was not statistically significantly affected by the outcome measure used to quantify resistance EI. Likewise, the secondary analysis, using EMG as the only outcome measure, showed a very large effect size comparable to that shown in the primary analysis. These results further emphasise the accuracy of RPE as a measure of both external and internal (physiological exertion) measures of exercise intensity, as was shown previously during team sports [3]. In aerobic exercise, Chen et al. [2] showed differences in validity coefficients based on the outcome measure used; additionally, there were contradictory moderator results dependent on the outcome measure used. It is possible that this shows a genuine improvement in RPE validity during resistance exercise over aerobic exercise when using certain outcome measures; conversely, this could simply be a consequence of the specific outcome measures and research articles included in each analysis.

#### EI Variable Manipulated

While the outcome measure used did not significantly affect validity, in the studies that used EI as the outcome measure, the variable used to modify EI did affect validity. The meta-regression analysis showed that significantly higher validity coefficients were shown in studies that manipulated workload (*p* < 0.001) or repetition time (*p* < 0.002), when compared to the use of total number of repetitions or rest interval time.

The accuracy of RPE to express changes in workload is well supported within the current literature. It has been suggested that RPE is sufficiently accurate to perceive differences in load at 20% intervals of 1RM, during dynamic biceps curls and knee extensions [16]; 10% differences in bench press power [66]; and 10-degree differences in knee angle during isometric wall squatting [31]. Fisher et al. [98] showed no differences in peak RPE or RPD between loads of 30% and 80% of 1RM in a test to fatigue. However, as both conditions were tested to fatigue, peak RPE and RPD would be expected to be the same (i.e., maximal) in both conditions. This was evident in a study by Vasquez et al. [83], that showed significant differences in RPE between different %1RM workloads at set repetition numbers but showed no significant differences between RPE at volitional fatigue. Genner and Weston [110] found that volume load (workload × total repetitions) shows stronger relationships with S-RPE than workload alone (%1RM); this result warrants further investigation.

The increased strength of the validity coefficients for repetition time could be related to the significantly greater coefficients seen with isometric exercise over the other forms of muscle contraction, as isometric contractions are normally sustained contractions and often controlled using contraction time; however, it is not clear whether the quantifiability of repetition time makes RPE during isometric contractions more accurate, or whether greater validity coefficients for isometric exercise have contributed to increased validity with repetition time modification.

The reduced validity shown with rest interval time should be interpreted with caution, as only two studies [48, 77] were included in the meta-analysis using rest time as the EI variable. Therefore, it is unclear whether manipulation of rest time really does produce lower validity coefficients, when compared to adjusting workload or repetition time, or whether there were just insufficient data at this time. Significant increases in S-RPE were shown with reduced rest interval when volume load was matched between conditions [111]. Senna et al. [112] and [113] showed inconsistent trends towards higher RPE ratings with lower rest times, however, these studies did not control the number of repetitions completed in each set, meaning significantly greater numbers of repetitions were achieved in the longer rest interval conditions. Additionally, Tibana et al. [114] showed no differences in RPE following exercise using either a 1.5- or 3-min rest period, but once again, these trials were both completed until volitional fatigue meaning that both conditions should elicit a maximal RPE response.

Gearhart et al. [115] explored differences in RPE during exercise with matched volume loads (tonnage); significantly higher RPE-AM ratings were seen with higher loads and fewer repetitions (5 reps @ 90% 1RM) compared to lighter weights with higher repetitions (15 reps @ 30% 1RM). Likewise, Kraft et al. [110] suggested that load had a greater effect on RPE than training volume changes. However, neither of these studies controlled the repetition time, meaning repetition time could have been increased in the higher load conditions without this increase being included in the exercise intensity calculation. In support of this, no significant differences were shown in RPE-O or S-RPE between conditions with matched volume load, but different workloads, when rest interval and repetition time were controlled [105]. In contrast, RPE was shown to increase with workload increase, despite matched volume load, during eccentric elbow flexion with standardised repetition and rest interval times. These conflicting findings and differences in procedures make interpretation difficult and prompt further investigation with full control over all EI variables [89].

Most of the studies included in the current review and meta-analysis, have manipulated one or more EI variable without measuring or controlling at least one of the other variables. Most commonly repetition time and rest interval are not measured, controlled, or reported. As shown in this analysis, changes in repetition time and rest interval time are correlated with changes in RPE. Additionally, increases in workload have been shown to inversely correlate with repetition velocity when repetition velocity/time are not controlled [19]. Ideally, studies looking to accurately manipulate exercise intensity should control and report all five of these EI variables, otherwise uncontrolled variables may change and thus magnify or nullify an expected change or create an unexpected change.

### RPE Reliability

Only 13 [4, 11, 28, 30–32, 37, 39, 55, 56, 59, 68, 76] of the 75 studies in the meta-analysis and 3 studies in the qualitative analysis [115–117] reported a measure of RPE repeatability. While single validity measurements are legitimate for RPE to be considered useful in a real-world exercise setting, especially when it is being used to prescribe exercise intensities, its results must be shown to be reliable between exercise sessions. In estimation mode, RPE-AM showed ‘good’ to ‘excellent’ reliability with intra-class correlation coefficients (ICC) of *r* = 0.67–0.96 [4, 28, 30–32, 37, 59, 76, 115], RPE-O showed ‘fair’ to ‘excellent’ reliability (*r* = 0.58–0.76) [4, 37], and S-RPE showed excellent reliability (*r* = 0.95; 95% CI = 0.90–0.97) [116]. These studies used various forms of exercise including isometric exercise [11, 30–32, 117], dynamic bench press and squatting [28], and mixed upper and lower body circuit training [39]. RPE was also shown to be reliable (*r* = 0.88; 95% CI = 0.89–0.91) during a home-based intervention [32], and when used in production mode (*r* = 0.69–0.95) [56].

Two studies [7, 68] showed lower ICC results than the rest of the included studies. The first [7] showed ICC results ranging from *r* = 0.07 to 0.80. The authors suggest that the lower scores were due to high inter-subject variance, and that while the ICC scores were low, agreement between the two sessions was much higher (60–90%). The second study [68] showed significantly lower RPE scores on the second testing day, when compared to the first day (*p* < 0.05) and showed ICC scores of *r* = − 0.05 to 0.46. It was argued that habituation with the exercise task, through additional testing days, could have reduced the between day differences as shown in previous studies [117].

Overall, these results suggest that RPE can be a reliable measure and prescribing tool; however, more research is required to confirm that RPE is reliable across a range of exercise intensities, participants, and exercise modes, and to elucidate which factors may positively and negatively affect its reliability.

### Additional Considerations for the Use of RPE

The current RPE literature has highlighted several possible confounding factors, that were outside the scope of this article’s statistical analysis but are worthy of consideration by practitioners using RPE in varied situations, environments, and with different populations; some of these considerations are outlined below:

Higher chronic perceived stress was associated with lower RPE and HR responses, in otherwise healthy participants, during strenuous resistance exercise [118]. Small but significant increases in RPE were shown following acute static stretching of the hamstrings [119]. No differences were shown in RPE or BLa between bodybuilders and powerlifters, during heavy resistance exercise with short rest intervals [55]. Likewise, the use of knee wraps during back squatting did not affect RPE results [47]. Conversely, blood flow restriction caused a significant increase in RPE at set exercise intensities [74]. Muscle activation and RPE were significantly increased when conducting push-up and leg raise exercises with an unstable base of support (i.e., a swiss ball) [120]. In support of this, whole body vibration at 40 Hz with a 4 mm amplitude, increased respiratory exchange ratio and RPE concurrently during back squatting. This was suggested to be due to an increase in type 2 fast twitch fibre activation for stabilisation [23].

In clinical or recently clinical populations, RPE was shown to be a good predictor of resistance exercise intensity in prostate cancer survivors [121], patients with multiple sclerosis produced statistically similar torque values at set RPE scores as healthy participants [122], and there was no significant difference in RPE during isometric endurance exercise in patients with chronic fatigue syndrome when compared to healthy participants [62]. More research is required to examine the usefulness of RPE in clinical populations, and the conditions and factors that may affect its accuracy.

### Limitations

There were several limitations in the present review. Firstly, we were only able to include studies written in the English language. Despite this, studies were included from a total of 11 countries, including 7 countries where English is not the first language: Brazil, Spain, Switzerland, Italy, Denmark, Taiwan, and France.

Secondly, some evidence of publication bias was present in the forest plot, indicating that some studies could exist that show less favourable results and have not been published. However, the Classic and Orwin’s fail safe tests showed that such a large number of non-significant studies would be required to change the validity level, and as such, it is extremely unlikely that this would have affected our results.

Finally, there was high between-study variance in the reported effect sizes, including a large amount of unexplained heterogeneity following the moderator analysis. This could largely be the consequence of the varied study designs, study populations, outcome measures and data reporting in the included studies. For example, exercise modalities including TheraBand exercise [4, 37], isometric wall squatting [30–32], and simulated manual work movements [53] were included. Additionally, we allowed any rating scale type for perceived pain or exertion. Inevitably, when including such a large number and variety of studies, greater between study variance is expected. Moreover, identifying, grouping, and coding these varied characteristics, in order to find moderators and explain heterogeneity, becomes increasingly difficult.

## Conclusions

In conclusion, these results suggest that RPE provides a valid measure of exercise intensity and physiological exertion during resistance exercise, with effect sizes comparable to or greater than those shown during aerobic exercise. As such, RPE may provide an easily accessible means of prescribing and monitoring resistance exercise training. Larger validity coefficients were seen in studies using greater workload ranges, isometric muscle action, and when EI was manipulated using workload or repetition time. Conversely, participant age, sex, training status, RPE scale used, and outcome measure used did not affect the validity coefficients reported. Further research is required to demonstrate RPE’s reliability and to further explore the possible moderating factors that could elicit different RPE results between populations and exercise types.

## Supplementary Information

**Additional file 1.** **Supplementary Table 1:** Study Methodological and Reporting Risk of Bias Assessment Tool for RPE Validation Studies. **Supplementary Figure 1:** Funnel Plot of Standard Error by Fisher’s Z. Clear diamond is the point estimate prior to any attempted corrections. Black diamond is the point estimate following Duval and Tweedie’s random-effects Trim and Fill adjustment. **Supplementary Table 2:** Univariate and multivariate meta-regression equations.

## Data Availability

All data supporting the results in this manuscript are available within the original manuscripts of the cited articles.

## Abbreviations

1RM: One-repetition maximum
BLa: Blood lactate
BP: Blood pressure
CI: Confidence intervals
EI: Exercise intensity
EMG: Electromyography
ERF: Estimated repetitions to failure
HR: Heart rate
IES: Isometric Exercise Scale
MVC: Maximal voluntary contraction
NRS: Numerical Rating Scale
OMNI-RES: OMNI Resistance Exercise Scale
PRISMA: Preferred reporting items for systematic reviews and meta-analyses
PTD: Perceived task duration
RES: Resistance exercise specific RPE
RIR: Repetitions in reserve
RPD: Rating of perceived discomfort or pain
RPE: Rating of perceived exertion
RPE-AM: Rating of perceived exertion for the active muscle(s)
RPE-O: Overall rating of perceived exertion
S-RPE: Sessional rating of perceived exertion
