# Supplementary material for: Convergent Validity of Ratings of Perceived Exertion During Resistance Exercise in Healthy Participants: A Systematic Review and Meta-Analysis
Source: Sports Med Open. 2022 Jan 8;8:2. doi: 10.1186/s40798-021-00386-8 (PMC8742800; doi:10.1186/s40798-021-00386-8)
Supplement: Supplementary file 1 — Additional file 1. Supplementary Table 1: Study Methodological and Reporting Risk of Bias Assessment Tool for RPE Validation Studies. Supplementary Figure 1: Funnel Plot of Standard Error by Fisher’s Z. Clear diamond is the point estimate prior to any attempted corrections. Black diamond is the point estimate following Duval and Tweedie’s random-effects Trim and Fill adjustment. Supplementary Table 2: Univariate and multivariate meta-regression equations. [file 40798_2021_386_MOESM1_ESM.pdf]

Criterion-Related Validity of Ratings of Perceived Exertion during Resistance Exercise in Healthy Participants: A Systematic Review and Meta-Analysis.

Sports Medicine

JOHN W. D. LEA[1], JAMIE M. O'DRISCOLL[1], SABINA HULBERT[1], JAMES SCALES[2] & JONATHAN D. WILES[1*]

[1] *School of Psychology and Life Sciences, Canterbury Christ Church University, Kent, CT1 1QU*
[2] *Institute of Population and Health Sciences, Queen Mary University of London, E1 4NS*
Corresponding author:* jim.wiles@canterbury.ac.uk, **Tel: 01227 922209

Supplementary Table 1: Study Methodological and Reporting Risk of Bias Assessment Tool for RPE Validation Studies

	Criterion	Explanation	Points for Inclusion	Score
1	Eligibility Criteria	Participant eligibility criteria is specified and fulfilled (including specific diagnostic test results)	1 Point	
2	Participant Information	Participant information is given, including age, sex, and training status	1 Point	
3	Power Analysis	Use of *a priori* power analysis/sample size calculation is acknowledged	1 Point	
4	Exercise Modality	Exercise type (dynamic, isometric etc.) and movement (squat, bench press etc.) is specified	1 Point	
5		Exercise intensity is specified (including load, number of sets, number of reps, rep time & rest time)	1 Point	
6	RPE Scale	Specify exact RPE scale used - including any modification	1 Point	
7	Anchoring Procedure	RPE instructions are specified	1 Point	
8		Anchoring procedures/methods are specified	1 Point	
9	Reliability Measure	A measure of repeatability/reliability was carried out and reported	1 Point	
			**Total (/9)**	

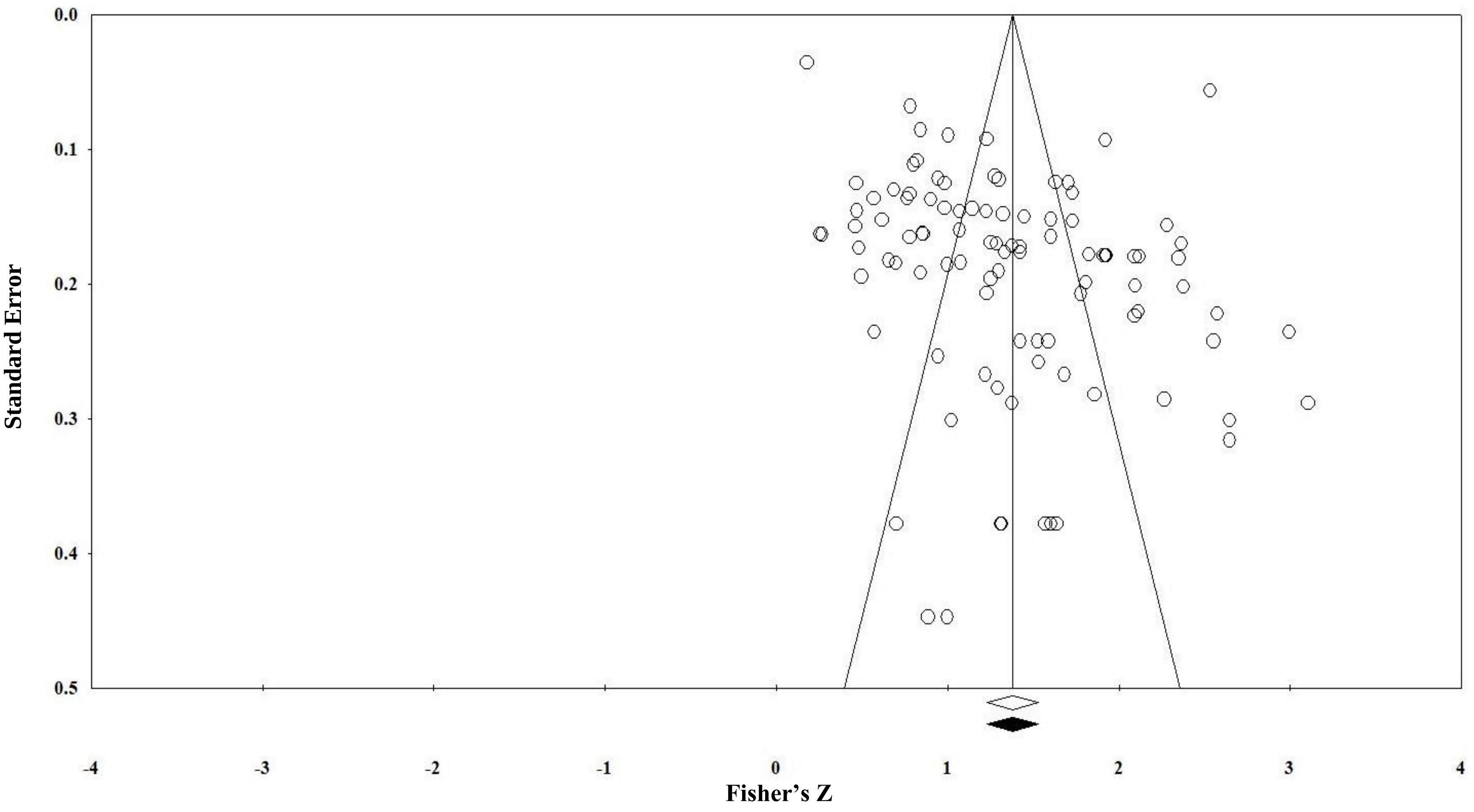

Supplementary Figure 1: Funnel Plot of Standard Error by Fisher's Z. Clear diamond is the point estimate prior to any attempted corrections. Black diamond is the point estimate following Duval and Tweedie's random-effects Trim and Fill adjustment.

Supplementary Table 2: Univariate and multivariate meta-regression equations

**Model**	**n**	**Covariate**	***β* (95% CI)**	***Q***	***I*²**	***τ*²**	***R*²**
**Univariate**							
Age	95	Intercept	1.2652 (0.9694 to 1.5610)***	0.87	94.3	0.42	0.00
		Age	0.0044 (-0.0048 to 0.0136)				
Sex	99	Intercept	1.3201 (1.0071 to 1.6332)***	0.18	95.5	0.47	0.11
		Male	0.0820 (-0.3010 to 0.4650)				
		Both	0.0596 (-0.3261 to 0.4453)				
Resistance Training Status	60	Intercept	1.4593 (1.1752 to 1.7434)***	5.53	92.8	0.34	0.18
		<6 month	-0.4385 (-0.9482 to 0.0712)				
		>6 month	-0.3470 (-0.7558 to 0.0618)				
		>1 Year	-0.0004 (-0.4255 to 0.4246)				
		Elite Level	-0.0082 (-0.9405 to 0.9241)				
Muscle Action	98	Intercept	1.2525 (1.0737 to 1.4314)***	7.01*	96.0	0.52	0.02
		Concentric	0.0730 (-0.9577 to 1.1037)				
		Eccentric	0.0350 (-1.4210 to 1.4909)				
		Isometric	0.4432 (0.1145 to 0.7719)**				
Body Segment		Intercept	1.2972 (1.0807 to 1.5138)***	1.72	95.7	0.49	0.10
		Lower	0.2054 (-0.1024 to 0.5132)				
		Whole	0.1278 (-0.4202 to 0.6757)				
Protocol		Intercept	1.4407 (0.8652 to 2.0163)***	0.03	94.1	0.40	0.00
		Intermittent	-0.0484 (-0.6408 to 0.5440)				
Workload Range	56	Intercept	0.5850 (0.2943 to 0.8758)***	43.8***	88.3	0.16	0.59
		Load Range	0.0165 (0.0116 to 0.0214)				
Scale Used	99	Intercept	1.3933 (1.0235 to 1.7631)***	9.46	95.9	0.50	0.04
		CR-10	-0.0192 (-0.4511 to 0.4126)				
		OMNI-RES	-0.1238 (-0.5745 to 0.3269)				
		ERF	0.2878 (-1.2438 to 1.8193)				
		Borg Words	0.9571 (-0.5251 to 2.4394)				
		IES	-0.1846 (-1.0875 to 0.7182)				
		NRS	0.6228 (-0.4679 to 1.7134)				
		PTD	1.7130 (0.1666 to 3.2594)				
		RES + RIR	-0.1941 (-1.3216 to 0.9334)				
Number of Points	91	Intercept	2.0902 (1.3458 to 2.8346)***	3.62	93.6	0.35	0.19
		Points	-0.0586 (-01190 to 0.0018)				
Fixed Maximum	98	Intercept	1.4190 (1.1418 to 1.6961)***	0.09	94.7	0.42	0.05
		Not fixed	-0.0491 (-0.3749 to 0.2768)				
Rating Mode	99	Intercept	1.3573 (1.1996 to 1.5150)***	0.71	95.6	0.50	0.05
		Production	0.1748 (-0.2307 to 0.5803)				
Rating Type	86	Intercept	1.4551 (1.2950 to 1.6153)	2.81	96.6	0.41	0.25
		RPE-O	-02413 (-0.6077 to 0.1250)				
		S-RPE	-0.5941 (-1.6027 to 0.4145)				
Outcome Measure		Intercept	1.3846 (1.2409 to 1.5284)***	1.31	94.9	0.44	0.16
		HR	-0.4429 (-1.5608 to 0.6750)				
		EMG	0.0908 (-0.4807 to 0.6624)				
		BLa	-0.4066 (-1.4395 to 0.6263)				

Supplementary Table 2 Continued: Univariate and multivariate meta-regression equations

**Model**	**n**	**Covariate**	***β* (95% CI)**	***Q***	***I*²**	***τ*²**	***R*²**
EI Variable Manipulated	83	Intercept	0.7986 (0.4582 to 1.1390)***	16.6***	93.9	0.36	0.14
		Workload	0.7093 (0.3324 to 1.0863)***				
		Rep Time	0.7563 (0.2452 to 1.2674)**				
		Rest Time	-0.3377 (-1.5986 to 0.9232)				
**Multivariate**	50	Intercept	0.5186 (0.1586 to 0.8786)**	38.5***	86.2	0.14	0.64
Sex		Male	0.1747 (-0.1091 to 0.4586)	2.35			
		Both	-0.0353 (-0.3461 to 0.2756)				
Rating Type		RPE-O	0.1091 (-0.2218 to 0.4400)	1.03			
		S-RPE	-0.4451 (-1.6121 to 0.7219)				
Workload Range		Load Range	0.0163 (0.0108 to 0.0217)***				

n number of cohorts included in analysis, *β* meta-regression coefficient, *Q* total weighted sum of squared differences between individual study effect sizes, I^2 percentage of between study variation that is due to heterogeneity, τ^2 between study variance, R^2 proportion of total variance explained by covariate model.
** p < 0.05
** p < 0.01
*** p < 0.001
